# Future prospects for new vaccines against sexually transmitted infections

**DOI:** 10.1097/QCO.0000000000000343

**Published:** 2017-01-05

**Authors:** Sami L. Gottlieb, Christine Johnston

**Affiliations:** a World Health Organization, Geneva, Switzerland; b University of Washington, Seattle, Washington, USA

**Keywords:** roadmap, sexually transmitted infections, sexually transmitted infection vaccine development, vaccines

## Abstract

**Purpose of review:**

This review provides an update on the need, development status, and important next steps for advancing development of vaccines against sexually transmitted infections (STIs), including herpes simplex virus (HSV), *Neisseria gonorrhoeae* (gonorrhea), *Chlamydia trachomatis* (chlamydia), and *Treponema pallidum* (syphilis).

**Recent findings:**

Global estimates suggest that more than a million STIs are acquired every day, and many new and emerging challenges to STI control highlight the critical need for development of new STI vaccines. Several therapeutic HSV-2 vaccine candidates are in Phase I/II clinical trials, and one subunit vaccine has shown sustained reductions in genital lesions and viral shedding, providing hope that an effective HSV vaccine is on the horizon. The first vaccine candidate for genital chlamydia infection has entered Phase I trials, and several more are in the pipeline. Use of novel technological approaches will likely see viable vaccine candidates for gonorrhea and syphilis in the future. The global STI vaccine roadmap outlines key activities to further advance STI vaccine development.

**Summary:**

Major progress is being made in addressing the large global unmet need for STI vaccines. With continued collaboration and support, these critically important vaccines for global sexual and reproductive health can become a reality.

## INTRODUCTION

To address the profound negative impact of sexually transmitted infections (STIs) on global sexual and reproductive health, in 1989 the World Health Organization (WHO) convened an expert advisory meeting to examine prospects for developing STI vaccines [1]. The participants’ assessment was rather disheartening. They noted that the only available STI vaccine at the time, against hepatitis B virus, had been only minimally implemented, and they considered the likelihood of developing a vaccine against human papillomavirus (HPV) to be very slim. Some experts felt that HPV vaccine development should not even be pursued [1]. Less than 15 years later, over half the world's infants had been immunized against hepatitis B, and the first HPV vaccines were shown to be efficacious in randomized controlled trials [2,3]. Currently, 95% of all countries include hepatitis B vaccination in their infant immunization programs, with 84% of newborns globally receiving three doses of the vaccine [3]. Because the first HPV vaccines were introduced in 2006, dramatic declines in HPV prevalence and HPV-related outcomes like genital warts have been observed in countries implementing the vaccine [4^▪^]. A new nine-valent HPV vaccine is highly efficacious in preventing HPV types causing 90% of cervical cancers [5^▪^], a disease that still affects more than half a million women a year, primarily in low-income and middle-income countries (LMICs) [6]. With financing support through Gavi, the Vaccine Alliance, HPV vaccines will soon be introduced across the hardest-hit countries, with the potential to avert millions of cervical cancer deaths.

These public health success stories provide inspiration for development of new STI vaccines. Although progress has been made over the past few decades in scaling up interventions to combat STIs, many existing and new challenges make the need for STI vaccines greater than ever [7]. For example, behavioral risk reduction efforts are cornerstones of STI prevention but have had limits in curbing STI transmission, and recent developments such as broadened use of pre-exposure prophylaxis for HIV prevention have paralleled increases in bacterial STIs in some settings [8]. STIs that have been easily treatable in the past are being threatened by new obstacles such as increasing resistance to cephalosporins for gonorrhea [9^▪▪^]. Lack of feasible, affordable STI diagnostic tests in many settings and the complexity and cost of screening programs have been longstanding barriers. Despite available prevention strategies, recent global estimates suggest that more than a million STIs are acquired every day [10^▪^,11^▪^]. An estimated 377 million new cases of *Chlamydia trachomatis* (chlamydia), *Neisseria gonorrhoeae* (gonorrhea), *Treponema pallidum* (syphilis), *Trichomonas vaginalis* (trichomoniasis), and herpes simplex virus type 2 (HSV-2) infections occurred in 2012 [10^▪^,11^▪^]. These infections result in a number of adverse outcomes, thus effectively addressing STIs can have a range of benefits, including: improving neonatal outcomes, for example, preventing mother-to-child transmission of syphilis [12^▪^]; decreasing the burden of infertility, of which chlamydia and gonorrhea are important causes [13^▪^,^14^]; reducing HIV transmission, as STIs such as HSV-2 lead to increased HIV acquisition and transmission [15]; combating antimicrobial resistance, a major concern for gonorrhea [16]; and supporting the health of young people, as the genital symptoms and psychosocial consequences of STIs have important effects on quality of life.

In 2013, WHO and the National Institutes of Allergy and Infectious Diseases (NIAID) held a second technical consultation on STI vaccines, almost 25 years after the first, and found that while challenges remain, the prospects for new STI vaccines are decidedly more promising [17]. Scientific advances, in conjunction with a confluence of global efforts related to improving sexual and reproductive health and reducing vaccine-preventable diseases [18,19], provide an opportune time to make these vaccines a reality. The latest STI vaccine consultation resulted in a global roadmap to advance STI vaccine development [20^▪▪^,^21^^▪▪^]. In this article, we review the need and development status for vaccines against HSV, chlamydia, gonorrhea, and syphilis, and discuss key STI vaccine roadmap activities to accelerate their advancement. 

**Box 1:**
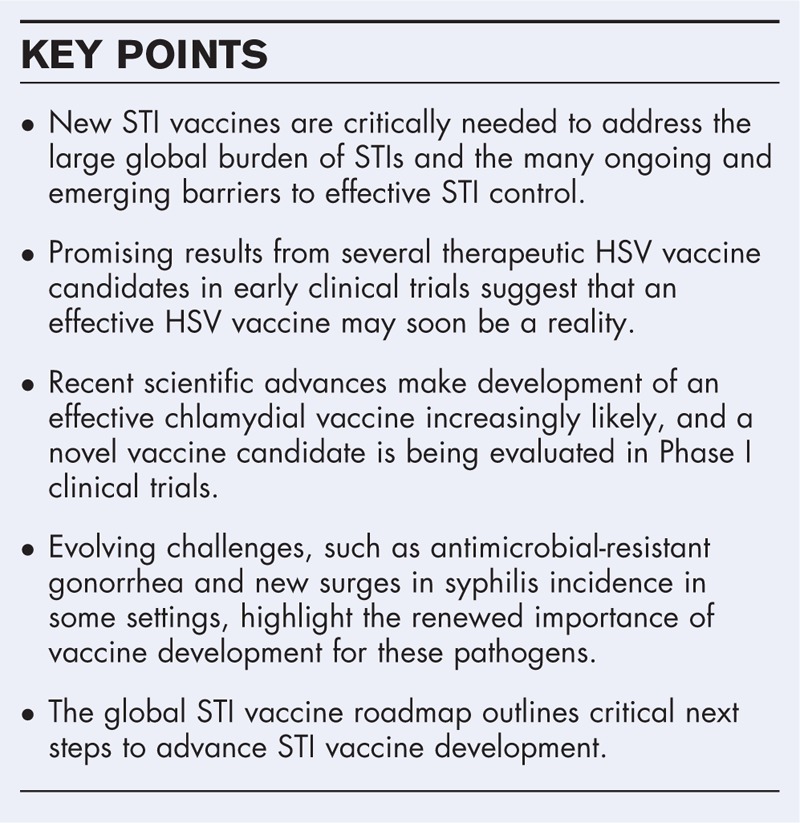
no caption available

## SEXUALLY TRANSMITTED INFECTION VACCINE DEVELOPMENT

**FIGURE 1:**
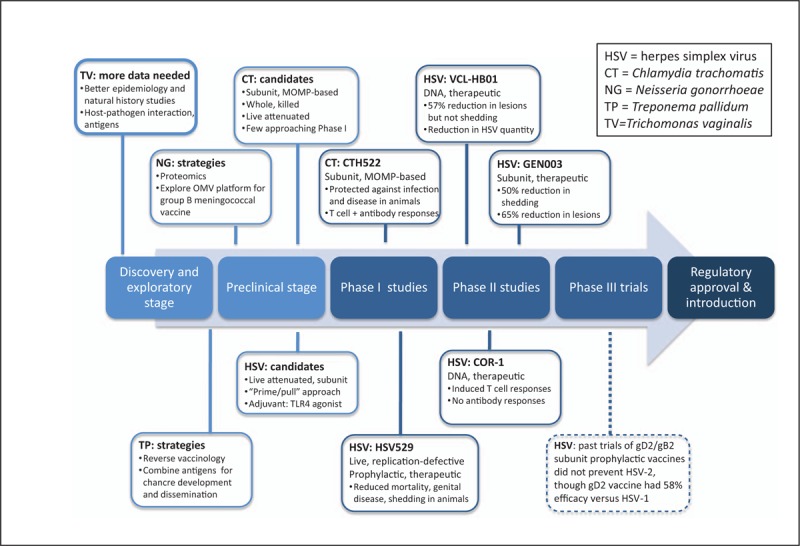
Research and development pipeline for STI vaccines. Five vaccine candidates, four for HSV [31,32^▪▪^,^33^,34^▪^,35^▪^] and one for CT [47^▪▪^,^54^], are in Phase I or II clinical studies. Multiple additional HSV and CT vaccine candidates are being evaluated in preclinical/animal studies; the main types of candidates or vaccine approaches are presented. Vaccine development for NG and TP is at earlier stages in the pathway; key strategies for developing viable candidates are highlighted. More data are needed to understand the path toward TV vaccine development. No current vaccine candidates are in Phase III clinical trials, but information from previous trials is provided (dotted line) [29]. MOMP, major outer membrane protein; OMV, outer membrane vesicle.

The current status of the development pathway for STI vaccines is shown in Fig. 1. HSV vaccine candidates are furthest along in the pathway, with several candidates in Phase I and II trials [22^▪^]. For years, genital chlamydia vaccine development was firmly in the preclinical stage; however, the first Phase I human clinical trials started in 2016, and others may soon follow [23]. Vaccine development for gonorrhea and syphilis is in earlier stages, but renewed commitment to these pathogens could result in new candidates over the next several years. Understanding prospects for vaccine development for trichomoniasis will require better epidemiologic, natural history, and basic science data, and will not be discussed in detail in this review [24].

### Herpes simplex virus

HSV-2 is the most common cause of genital herpes, with an estimated 417 million people aged 14–49 infected worldwide [10^▪^]. In addition, 140 million adults are estimated to have genital infection with HSV-1, which is often acquired orally in childhood but is now an important cause of genital herpes in many high-income countries (HICs) [25^▪^]. Genital HSV infection leads to chronic infection with a lifelong reservoir in the sacral ganglia. Viral reactivation occurs frequently, particularly for HSV-2, leading to recurrent genital ulcers or asymptomatic viral shedding at the genital skin or mucosa, during which HSV can be transmitted. A major negative public health consequence of HSV-2 infection is its role in propagating the HIV epidemic, as chronic genital inflammation from HSV-2 increases HIV acquisition risk by two-fold to three-fold [15]. In Kenya, the estimated population attributable fraction of HIV infection due to HSV-2 is 48% [26]. Mother-to-child HSV transmission causing neonatal herpes is rare but often leads to infant death or devastating neurologic damage. Prevention tools including antivirals and condoms can partially reduce HSV transmission risk for individuals, but no method provides adequate protection, and an HSV vaccine is a much needed prevention strategy.

Two strategies are being pursued for HSV-2 vaccine development. The classic approach uses a prophylactic vaccine targeting people who are not infected to prevent HSV acquisition. Alternatively, a therapeutic vaccine is designed for people who already have HSV-2 infection to reduce shedding and recurrences. Whether these two approaches will require different types of immunologic responses is unknown. Both neutralizing antibody responses and cell-mediated immunity may be important for a prophylactic vaccine [27], whereas stimulation of recently described tissue-resident memory T cells is likely essential for therapeutic vaccination [28]. Several adjuvanted subunit vaccines targeting HSV glycoprotein D2 (gD2) with or without glycoprotein B2 (gB2) have been tested in Phase III clinical trials as prophylactic vaccines. Despite eliciting strong neutralizing antibody responses, none prevented HSV-2 acquisition. The most recent trial (Herpevac), which tested an adjuvanted gD2 vaccine in HSV-1/HSV-2-seronegative women, failed to prevent symptomatic genital herpes disease overall [29]. However, this vaccine did prevent genital herpes due to HSV-1, with a vaccine efficacy of 58%. Increasing antibody titers to gD2 were associated with increased vaccine efficacy against HSV-1, providing the first immune correlate of protection [30]. Although these secondary findings are promising, investment in prophylactic HSV vaccine development has declined following the results of these studies.

In contrast, the past 5 years has seen intense interest in development of a therapeutic HSV-2 vaccine, with multiple novel platforms and adjuvants under evaluation (Fig. 1). Three such candidates are currently in Phase II trials. The most advanced, GEN-003, is a subunit vaccine containing a deletion mutant of gD2 and a portion of infected cell protein 4 (ICP4), with Matrix-M2 adjuvant. In a Phase I/IIa study, participants receiving the most efficacious dose of GEN-003 had a 50% decrease in viral shedding and a 65% decrease in days with genital lesions, persisting for 12 months postvaccination [31]. T cell and antibody responses to gD2 and ICP4 also remained elevated for 12 months [32^▪▪^]. A second Phase II trial is evaluating an optimized formulation of GEN-003. Another candidate, VCL-HB01, is a DNA vaccine containing two codon-optimized genes (gD2+VP11/12) with Vaxfectin adjuvant. In a Phase I/II study among HSV-2-seropositive people, VCL-HB01 did not meet the primary endpoint of decreased HSV shedding, but vaccine recipients had a 57% decrease in lesion frequency at 9 months and reduction in quantity of virus detected [33]. The vaccine also induced UL46-specific T cell responses. Another DNA vaccine candidate, COR-1, contains codon-optimized gD2 and ubiquitin-fused truncated gD2 to enhance generation of cytotoxic T cells. COR-1 was safe in HSV-1/2-seronegative participants in a Phase I study and induced gD2-specific T cell but not antibody responses [34^▪^]. Results of a Phase II evaluation of COR-1 are forthcoming.

In earlier stages of development, HSV529 is a novel live, replication-defective HSV-2 with deletions in UL5 and UL29, which reduced mortality, genital disease severity, and viral shedding in animal models [35^▪^]. Phase I testing of HSV529 in HSV-2-seropositive and HSV-2-seronegative people and evaluation of genital immune responses is ongoing. All of these vaccine studies are providing valuable information about immunity to genital HSV and insights into optimal trial design for future Phase III trials. Current vaccine candidates target HSV-2, but identification of cross-reactive epitopes against HSV-1 and HSV-2 raise the possibility that a vaccine targeting both HSV types could be developed [36^▪^]. In addition, genomic sequencing of HSV-2 from different regions, revealing many highly conserved antigens, could ensure a geographically unrestricted vaccine [37].

### Chlamydia trachomatis

Genital chlamydia infection is a concern in all world regions, with an estimated 131 million incident cases globally in 2012 [11^▪^]. Young people, and adolescents in particular, are disproportionately affected [38]. Without treatment, chlamydia can ascend to the upper genital tract in women to cause acute pelvic inflammatory disease (PID), which can in turn lead to longer-term complications including tubal factor infertility, ectopic pregnancy, and chronic pelvic pain. The vast majority of chlamydia infections are asymptomatic and because tests are lacking in many settings, especially in LMICs, most infections are not diagnosed. Even when tests are available, chlamydia screening programs have had difficulty achieving high coverage levels in HICs [39], do not appear to have reduced chlamydia transmission [40], and even in the best case scenario might be expected to prevent only about 60% of chlamydia-related PID [41^▪^]. Recent comprehensive models suggest that every 1000 chlamydia infections result in five women with tubal factor infertility in HICs [41^▪^]. Given the estimated 68 million chlamydial infections among women each year [11^▪^], the global burden of chlamydia-related sequelae is likely substantial.

Fortunately, development of chlamydia vaccines is advancing. A wealth of animal data and several human studies show that natural infection results in short-lived partial protective immunity [42,43]. In one study, women whose chlamydial infections cleared spontaneously between testing and treatment were less likely to become re-infected on follow-up [44]. The precise mechanisms of immunity are not completely understood, but interferon-γ (IFN-γ)-producing CD4^+^ T cells play a critical role, and tissue-resident memory T cells may be particularly important for vaccine development [45,46^▪▪^]. Antibodies play some role, whether from enhancement of Th1 effector responses or direct pathogen neutralization [47^▪▪^]. Novel antigens for chlamydial vaccine development have been identified through reverse vaccinology approaches, which start with computer-based analysis of the whole genome to predict likely vaccine targets, and immunoproteomics, which involves high-throughput evaluation of large protein sets to investigate antigens interacting with the host immune system [48,49^▪^,^50^]. Immune profiling of well-characterized clinical cohorts has further clarified potential vaccine targets [51^▪^]. Genetic manipulation of *C. trachomatis*[52], combined with work on novel adjuvants and delivery systems [53^▪^], is also expanding the list of vaccine candidates.

The main vaccine approaches include subunit vaccines based on the chlamydial major outer membrane protein (MOMP), whole inactivated vaccines, and live attenuated vaccines. A recombinant MOMP subunit vaccine candidate promoted strong neutralizing antibody titers and Th1 responses and showed protection against vaginal chlamydial infection in mini-pigs and against upper genital tract disease in mice [47^▪▪^,^54^]. This candidate entered human Phase I clinical trials in 2016 [23]. Combination of MOMP with polymorphic membrane proteins identified by immunoproteomics is another promising approach [49^▪^]. A major advance in the field has been the ability to generate vaccine-induced seeding of genital mucosa with CD4^+^ tissue-resident memory T cells, which was the key to long-lived protection against chlamydial infection in mice [46^▪▪^]. This was achieved using mucosal immunization with UV-inactivated *C. trachomatis* combined with a novel nanoparticle-based adjuvant [46^▪▪^]. An attenuated plasmid-free chlamydial strain being evaluated as a vaccine against ocular *C. trachomatis* infection (trachoma) may also inform vaccine development for genital infection [55].

### Neisseria gonorrhoeae

STI control strategies based on prompt antibiotic treatment for symptomatic patients and focused partner management have been effective at reducing the incidence of gonorrhea [56]. However, increasing evidence of resistance to cephalosporins, the only remaining first-line drugs for gonorrhea [57], reports of multidrug resistance [58], and progressive resistance to sequential antibiotics [16] create an urgent need for new prevention strategies. A high burden of gonorrhea exists in many LMICs, with an estimated 78 million incident infections globally in 2012 [11^▪^]. In addition, there has been a resurgence of gonorrhea incidence in many HICs, especially among men who have sex with men [8]. Genital gonorrhea has adverse outcomes similar to those of chlamydia, such as PID and infertility, but there is even more limited understanding of the burden of gonorrhea-related sequelae globally. Thus, the potential threat of untreatable gonorrhea with expanding antimicrobial resistance makes vaccine development crucial.

Many biological challenges exist to gonococcal vaccine development. There is no naturally acquired immunity to the infection; *N. gonorrhoeae* has a highly antigenically variable surface and is well adapted to evade host responses, and robust animal models to study the infection are limited [59]. Multiple potential gonorrhea vaccine targets have been identified based on their relative antigenic conservation and stability among strains [60^▪^], but these have not yet yielded viable vaccine candidates. However, more sophisticated mouse models are now available to evaluate immune responses and disease in a way that more closely mimics human infection [61^▪^]. In addition, new high-throughput techniques such as proteome mining, which uses bioinformatics to select proteins with desired characteristics from large datasets, have narrowed the search for promising antigenic targets [62^▪^]. Translational studies applying molecular techniques to clinical specimens allow assessment of genes expressed during gonococcal infection [63].

A promising development for gonococcal vaccine discovery relates to existing vaccines against another *Neisseria* species, in particular the group B meningococcal vaccine using the outer membrane vesicle (OMV) antigen presentation strategy. *N. gonorrhoeae* and *N. meningitidis* share 80–90% homology of primary sequences and thus some level of cross-protection is plausible. A recent case–control study in New Zealand, wherein group B OMV meningococcal vaccine has been used for years, suggests a decrease in gonorrhea infection in those who have received the OMV meningococcal vaccination [64]. Expanding upon this platform could provide a template for a successful gonococcal vaccine or a broader *Neisseria* vaccine incorporating gonococcal antigens.

### Treponema pallidum

Syphilis incidence has decreased globally [11^▪^] but remains an important cause of fetal and neonatal mortality in many LMICs, with over 200 000 fetal and neonatal deaths estimated annually [12^▪^,^65^]. In addition, in several HICs with very low syphilis rates, there has been a resurgence in syphilis incidence, especially among men who have sex with men [66]. A main goal of the new Global Health Sector Strategy for STIs, 2016–2021, is to reduce global syphilis incidence by 90% by 2030 [67^▪^]. However, most syphilis control programs in LMICs focus on preventing congenital syphilis through antenatal screening and treatment. It has been less clear how to reduce population-wide incidence, especially with barriers to effective partner treatment programs in resource-poor settings. These challenges are compounded by new concerns about supply chain shortages of benzathine penicillin, the only first-line treatment for syphilis [68]. These considerations have led to renewed interest in syphilis vaccine development.

Few investigators work on syphilis vaccine development, primarily due to lack of consistent funding and difficulties using the existing rabbit model of infection [69]. In the early 1970s, rabbits given multiple injections of irradiated *T. pallidum* over many weeks became immune to disease on subsequent challenge [70]. Although the immunization regimen used was not tenable for humans, this provided proof of concept that protection against syphilis is possible. Current efforts focus on reverse vaccinology and targeted functional studies to identify antigens important for pathogen–host interactions and pathogenesis [71,72]. Sequencing circulating syphilis strains provides additional information on potential cross-protection across selected targets [73]. The challenge now is to generate the right combination of these potential vaccine targets, with appropriate adjuvants, to develop a viable syphilis vaccine candidate [74^▪^].

## NEXT STEPS: THE ROADMAP FOR SEXUALLY TRANSMITTED INFECTION VACCINE DEVELOPMENT

Vaccine development progresses through a defined set of stages, often over many years (Fig. 1). The process is expensive and thus risky for vaccine developers, but several factors can help ‘de-risk’ the process to facilitate vaccine development, for example, a clearly defined market for the vaccine or an advance in technology. The global STI vaccine roadmap outlines six main areas to accelerate vaccine development: first, obtaining better epidemiologic data on infection and sequelae; second, modeling the theoretical impact of STI vaccines; third, advancing basic science and translational research; fourth, defining preferred product characteristics (PPCs); fifth, facilitating clinical evaluation and vaccine introduction; and finally, encouraging investment in STI vaccine development [20^▪▪^]. Within each area, the roadmap delineates key action steps, many of which can be pursued in parallel to catalyze vaccine development [20^▪▪^,^21^^▪▪^].

Table 1 shows selected roadmap activities that are critical for STI vaccine development, all of which help encourage investment in STI vaccines, the final action area. For example, a key activity for obtaining better epidemiologic data is research on the burden of chlamydia-associated and gonorrhea-associated PID, infertility, and ectopic pregnancy, especially in LMICs. Newer serologic tests for *C. trachomatis* may facilitate assessing the population attributable fraction of these outcomes due to chlamydia [75^▪^]. The Child Health and Mortality Prevention Surveillance (CHAMPS) network, which will explore causes of neonatal deaths in developing countries, will collect much needed data on fatal neonatal syphilis and HSV infections [76]. Modeling theoretical impact is essential for all vaccines and should consider different epidemiologic and economic settings and include cost-effectiveness analyses. Existing models for HSV and chlamydia vaccines demonstrate that even vaccines with modest efficacy could have an important health impact [77,78] and could be cost-effective [79]. A 2015 WHO HSV vaccine modeling meeting stressed including HSV-associated HIV incidence and neonatal herpes as outcomes and incorporating protection against or attenuation due to HSV-1 infection in updated models. Complementary models can explore the added benefit of a gonorrhea vaccine under differing levels of antimicrobial resistance, and the potential for a vaccine to thwart such resistance.

**Table 1:** Key STI vaccine roadmap activities

	All^a^	HSV	Chlamydia	Gonorrhea	Syphilis
Obtaining better epidemiologic data on infection and disease	Improve global and regional estimates of STI burden;Explore use of existing STI data from clinical trials, studies;Develop and validate STI diagnostic tests for LMICs;Refine quality of life estimates;Obtain data on STI costs, especially in LMICs	Obtain primary data on neonatal herpes in LMICs;Update PAF estimates of HIV due to HSV-2;Clarify burden of genital ulcer disease and effects on quality of life	Refine estimates of CT-related PID, infertility, especially in LMICs;Assess PAF of outcomes due to CT; explore use of newer serologic tests	Refine estimates of NG-related PID, infertility, especially in LMICs;Monitor epidemiology of NG AMR	Assess syphilis-related fetal and neonatal deaths in CHAMPS network;Monitor new increases in syphilis incidence in HICs
Modelling theoretical vaccine impact	Develop models for vaccine impact in diverse settings;Determine key data gaps to drive models;Assess impact under different assumptions and scenarios;Include costs;Use models to guide key vaccine characteristics	Include broader HSV outcomes, including HSV-associated HIV incidence;Model HSV-1 as outcome and potential modifier of vaccine impact on HSV-2;Evaluate therapeutic vs. prophylactic vaccines	Update models to obtain better data on outcomes;Model vaccine impact and cost-effectiveness in LMICs, settings without screening	Model vaccine impact under differing levels of NG AMR;Model impact of vaccine on development of AMR	Assess vaccine impact vs. antenatal screening for congenital syphilis elimination;Assess vaccine impact vs. antenatal screening for population transmission
Advancing basic science and translational research	Assess correlates of protection;Define best animal models;Use genomic, proteomic approaches to screen antigens;Explore immune responses in well defined clinical cohorts;Optimize adjuvants and delivery systems;Understand role of hormones and microbiome	Determine approaches to generate tissue-resident memory T cells for HSV;Test new adjuvant and antigen combinations	Incorporate approaches to generate tissue-resident memory T cells;Evaluate antigens identified by reverse vaccinology, proteomics;Test new adjuvant and antigen combinations	Explore use of group B meningococcal OMV vaccine platforms for NG;Examine use of human male urethral challenge model to assess correlates of protection	Study host–pathogen interactions and immune evasion mechanisms;Identify cross-protective antigens via sequencing;Combine antigens critical for chancre formation and for dissemination
Defining preferred product characteristics (PPCs)	Outline desired vaccine goals and indications, especially for LMICs;Define acceptable levels of efficacy and safety to reach public health goals;Clarify target populations;Describe possible vaccination strategies	Define goal, indications: infection vs. disease;Prevention of HSV-2 only vs. HSV-2 + HSV-1;Clarify: prophylactic vs. therapeutic vaccine;Set minimum efficacy requirements;Define target population: adolescent vs. infant	Outline infection vs. disease indications;Define target population: girls only vs. girls + boys;Consider efficacy needs: prevention at cervix vs. ascension to upper genital tract	Consider role of AMR in determining PPCs;Determine whether target populations vary by setting (HICs/LMICs);Consider combination with another vaccine (e.g., CT, meningococcal)	Determine whether target populations vary by setting (HICs/LMICs);Assess possibilities for use as maternal vaccine, safety requirements
Facilitating clinical evaluation and vaccine introduction	Reach consensus on clinical trial endpoints;Improve measuring endpoints, validating surrogate endpoints;Strengthen trial design, sites;Involve regulators, determine regulatory route to licensure;Promote Phase I evaluation;Establish systems to monitor outcomes in advance	Define clinical endpoints: infection vs. genital ulcer disease, role of shedding;Understand potential risks/benefits of evaluation in high HIV prevalence areas	Define clinical endpoints: infection vs. PID;Develop biomarkers, radiological tests, etc. for upper tract disease	Define clinical endpoints: infection vs. PID;Develop biomarkers, radiological tests, etc. for upper tract disease;Explore use of human male urethral challenge model in Phase I/II trials	Determine trial design, population, and setting;Assess possible evaluation as maternal immunization

AMR, antimicrobial resistance; CHAMPS, Child Health and Mortality Prevention Surveillance; CT, *Chlamydia trachomatis*; HICs, high-income countries; HSV, herpes simplex virus; LMICs, low middle income countries; NG, *Neisseria gonorrhoeae*; OMV, outer membrane vesicle; PAF, population attributable fraction; PID, pelvic inflammatory disease; STI, sexually transmitted infection.

^a^Includes trichomoniasis, for which better epidemiologic data on burden of infection, natural history, and related disease, and additional basic science data on pathogenesis and the host immune responses to *Trichomonas vaginalis* are critical first steps for vaccine development.

To advance basic science, NIAID has held workshops on HSV, chlamydia, and gonorrhea vaccine development [61^▪^,^80^]. These workshops have brought scientists together to identify, standardize, and share reagents, immunogens, assays, and animal models to accelerate moving vaccine candidates into clinical evaluation. Important next steps include capitalizing on novel scientific advances, such as exploiting the importance of tissue-resident memory T cells in preventing HSV-2 and chlamydia infection [46^▪▪^], and using novel models to evaluate vaccine candidates and conduct translational work, such as the human male urethral challenge model for gonorrhea [81]. It will be important to explore vaccine mechanisms and adjuvants used for other pathogens to find potential uses with STI vaccines, for example the OMV group B meningococcal vaccine for gonorrhea [65].

PPCs reflect WHO guidance on desired parameters of a vaccine to meet priority public health goals, primarily for LMICs [82]. PPCs describe characteristics such as vaccine goals, target groups, immunization strategies, and data needed to ensure safety and efficacy. For example, PPCs may define whether the vaccine goal is prevention of morbidity or infection, and the minimum efficacy required to achieve a public health benefit. PPCs are now being developed for HSV vaccines. Important considerations include whether prophylactic or therapeutic vaccines are desired for LMICs, especially those with high HIV prevalence, and whether HSV vaccines should target HSV-2 only or both HSV types, which may influence the target age for immunization. Consensus building around clinical endpoints and trial design is essential for all vaccines, but may be particularly important for chlamydia vaccine. The ultimate goal is to decrease upper genital tract sequelae; however, there are challenges with measuring PID as a clinical endpoint, an insensitive, nonspecific, and multifactorial diagnosis [13^▪^]. A critical need is better measures of tubal involvement and surrogate endpoints, including biomarkers or radiologic measures of upper genital tract infection, inflammation, and damage.

Together, the roadmap activities can help generate comprehensive business cases to outline the public health rationale for each vaccine and inform decision-making, which are critical for needed investment in the field. Clearly defining disease burden and costs enables modeling of vaccine impact and cost-effectiveness and determines the vaccine market. This can be weighed against vaccine development costs, which depend on technology and the desired characteristics of the vaccine. A heightened awareness of the need for STI vaccines will also be paramount for building on current progress, as will innovative product development partnerships, which have been successful for vaccines against other neglected diseases [83]. Toward this end, an STI vaccine initiative is envisioned to bring together public health institutions, academia, donor agencies, and industry to facilitate collaboration and implement the STI vaccine roadmap [21^▪▪^,^84^].

## CONCLUSION

Twenty-five years ago, the outlook for development and implementation of the first STI vaccines, against HPV and hepatitis B, seemed bleak [1]. Yet in the ensuing years, these vaccines became major advances for global public health. Following on these successes, development of a new generation of STI vaccines is now within reach. Multiple promising vaccine candidates in early clinical trials provide real hope that a therapeutic HSV vaccine is on the horizon. The first new chlamydia vaccine candidate has entered Phase I trials, and several more candidates may soon follow. Emerging challenges to STI control, such as antimicrobial resistance for gonorrhea and new syphilis outbreaks, create a new urgency for these vaccines. Although challenges remain, the STI vaccine roadmap provides a guide for capitalizing on the momentum to develop STI vaccines [20^▪▪^,^21^^▪▪^]. With continued support and collaboration, these much needed vaccines can be made a reality.

## Acknowledgements

The authors would like to thank Drs Carolyn Deal, Nathalie Broutet, and Birgitte Giersing for helpful discussions about STI vaccine development.

### Financial support and sponsorship

S.L.G. is a salaried employee of the World Health Organization. C.J.'s work was supported by the National Institutes of Health (NIH AI030731).

### Conflicts of interest

S.L.G. reports no potential conflicts of interest. The University of Washington has received funds for C.J. to conduct research sponsored by the following companies as a principal or co-investigator: Agenus, Genocea, Vical, Gilead, AiCuris, and Sanofi.

Disclaimer: S.L.G. is a staff member of the World Health Organization. The author alone is responsible for the views expressed in this article, which do not necessarily represent the decisions or policies of the World Health Organization.

## REFERENCES AND RECOMMENDED READING

Papers of particular interest, published within the annual period of review, have been highlighted as:

▪ of special interest
▪▪ of outstanding interest
